# TIE1 promotes cervical cancer progression via Basigin-matrix metalloproteinase axis

**DOI:** 10.7150/ijbs.93667

**Published:** 2024-04-08

**Authors:** Pan Liu, Lisha Xie, Qiulei Wu, Lin Huang, Xiaoli Liu, Wenhan Li, Jing Cai, Zehua Wang, Ping Yang, Liqiong Cai

**Affiliations:** 1 Department of Obstetrics and Gynecology, Union Hospital, Tongji Medical College, Huazhong University of Science and Technology, Wuhan 430022, China.; 2 Department of Obstetrics and Gynecology, First Affiliated Hospital, School of Medicine, Shihezi University, Shihezi 832003, China.

**Keywords:** TIE1, Basigin, matrix metalloproteinases, cervical cancer.

## Abstract

**Background:** Tyrosine kinase with immunoglobulin and EGF-like domains 1 (TIE1) is known as an orphan receptor prominently expressed in endothelial cells and participates in angiogenesis by regulating TIE2 activity. Our previous study demonstrated elevated TIE1 expression in cervical cancer cells. However, the role of TIE1 in cervical cancer progression, metastasis and treatment remains elusive.

**Methods:** Immunohistochemistry staining for TIE1 and Basigin was performed in 135 human cervical cancer tissues. Overexpressing vectors and siRNAs were used to manipulate gene expression in tumor cells. Colony formation, wound healing, and transwell assays were used to assess cervical cancer cell proliferation and migration *in vitro*. Subcutaneous xenograft tumor and lung metastasis mouse models were established to examine tumor growth and metastasis. Co-Immunoprecipitation and Mass Spectrometry were applied to explore the proteins binding to TIE1. Immunoprecipitation and immunofluorescence staining were used to verify the interaction between TIE1 and Basigin. Cycloheximide chase assay and MG132 treatment were conducted to analyze protein stability.

**Results:** High TIE1 expression was associated with poor survival in cervical cancer patients. TIE1 overexpression promoted the proliferation, migration and invasion of cervical cancer cells *in vitro*, as well as tumor growth and metastasis *in vivo*. In addition, Basigin, a transmembrane glycoprotein, was identified as a TIE1 binding protein, suggesting a pivotal role in matrix metalloproteinase regulation, angiogenesis, cell adhesion, and immune responses. Knockdown of Basigin or treatment with the Basigin inhibitor AC-73 reversed the tumor-promoting effect of TIE1 *in vitro* and *in vivo*. Furthermore, we found that TIE1 was able to interact with and stabilize the Basigin protein and stimulate the Basigin-matrix metalloproteinase axis.

**Conclusion:** TIE1 expression in cervical cells exerts a tumor-promoting effect, which is at least in part dependent on its interaction with Basigin. These findings have revealed a TIE2-independent mechanism of TIE1, which may provide a new biomarker for cervical cancer progression, and a potential therapeutic target for the treatment of cervical cancer patients.

## Introduction

Over the past few decades, the prevalence and mortality of cervical cancer have decreased in developed countries due to increased cervical cancer screening. However, it remains the second most commonly diagnosed and deadly female cancer in developing countries 1. Patients with early or locally advanced disease typically have a favorable prognosis, with an overall five-year survival rate of over 80% or approximately 70%, respectively 2. However, for metastatic or recurrent cervical cancer, the five-year survival rate is less than 20% 3. Therefore, it is still urgent to identify critical molecular mechanisms involved in cervical cancer progression.

Tyrosine kinases with immunoglobulin and EGF-like domains (TIEs) are a class of receptor tyrosine kinases that are mainly expressed in endothelial cells and are essential for promoting angiogenesis and maintaining vascular integrity 4, 5. Although TIE1 and TIE2 have great structural homology, only TIE2 can interact with its ligand angiopoietins. TIE1 has long been regarded as an orphan receptor that acts principally by forming a heterodimer with TIE2 to regulate its activity 6. Our previous study demonstrated elevated TIE1 expression in cervical cancer epithelial cells relative to normal cervical epithelium 7. However, the role and mechanism of TIE1 in cervical cancer progression remain elusive.

In the present study, we found that Basigin was a target of TIE1. Basigin, a transmembrane glycoprotein which is highly expressed in various human cancers. It has been initially recognized as a regulator of matrix metalloproteinases (MMPs) 8, playing a vital role in promoting tumor invasion and metastasis by degrading the extracellular matrix or indirectly regulating related cytokines 9, 10. One mechanism by which Basigin promotes MMPs expression is through forming a homodimer, activating the downstream MAPK/ERK signalling pathway 11. The tumor-promoting role of Basigin has been demonstrated in multiple cancers, such as lung cancer, hepatocellular carcinoma and colorectal cancer 12-14. In cervical cancer, high expression of Basigin is closely associated with poor tumor differentiation and metastasis 15-17. Here, we uncovered a novel molecular mechanism by which TIE1 promotes cervical cancer progression, independent of TIE2. The crucial role of TIE1 is mediated by binding and stabilizing Basigin to increase the expression of MMP2 and MMP9. Additionally, TIE1 has been shown to be associated with a poor prognosis in cervical cancer. Thus, we identified TIE1 as a prognostic factor and provided a powerful molecular basis for TIE1-mediated anti-cervical cancer therapy.

## Materials and methods

### Patients and specimens

This study complied with the Declaration of Helsinki and was approved by the Ethics Committee of Tongji Medical College, Huazhong University of Science and Technology (IORG No: IORG0003571). Informed consent was obtained from all patients. We collected 135 cervical cancer samples from patients who underwent surgery without prior radiotherapy, chemotherapy or targeted therapy in the Department of Obstetrics and Gynecology, Wuhan Union Hospital, Tongji Medical College (Wuhan, China) between January 1, 2013, and January 1, 2017. The inclusion criteria, clinicopathologic characteristics, and follow-up data collection were consistent with those in our previous study 18.

### Immunohistochemistry (IHC)

A tissue microarray was constructed for IHC staining. The IHC process has been previously described 18. The antibodies used are listed in Table S2. The IHC results were scored by multiplying the staining intensity (0 for no signal, 1 for weak, 2 for moderate, 3 for strong) and the proportion of positive tumor cells 19. The results of IHC were assessed by two specialized pathologists.

### Cell lines and cell culture

Human cervical cancer cell lines (HeLa and SiHa) were purchased from the China Center for Type Culture Collection (Wuhan, China). Cells were cultured in DMEM (Basal media, Shanghai, China) supplemented with 10% fetal bovine serum (GIBCO, America) in a humidified atmosphere with 5% CO_2_ at 37℃. All the cell lines were passaged no more than 15 times and authenticated by short tandem repeat (STR) genotyping. All cell lines were verified to be free of mycoplasma contamination.

### Transfection

All siRNAs were purchased from Tsingke (Wuhan, China), and transfections were performed using Lipofectamine 3000 (Thermo Fisher, USA) following the manufacturer's instructions. The siRNA sequences used in this study are described in Table S3. HeLa and SiHa cell lines that stably expressed TIE1 were generated using the GV492 lentiviral vector (GENECHEM, China). Cells were infected with lentiviral particles, and the transduced cells were then selected by puromycin (2 μg/mL) for at least 5 days.

### Colony formation assays

Cells were seeded in six-well plates (600 cells per well) and cultured for 2 weeks. Cells were then fixed with paraformaldehyde (4%) for 15 min, stained with 0.1% crystal violet for 30 min, and photographed using a digital scanner. The experiment was performed independently in triplicate.

### Wound healing and transwell assays

Wound healing and transwell migration assays were performed to evaluate cell migration ability, while transwell invasion assays were used to assess cell invasion ability. Detailed procedures are described in the Supplementary Methods. Each experiment was repeated in triplicate.

### Co-Immunoprecipitation and Mass Spectrometry (Co-IP/MS)

Detailed descriptions of the co-immunoprecipitation and mass spectrometry (Co-IP/MS) procedure can be found in the Supplementary Methods.

### Western blotting and immunofluorescence staining

Western blotting and immunofluorescence staining assays were performed as described previously 20. The quantification of Western blotting was normalized to GAPDH levels. Immunofluorescence staining images were performed by Confocal microscope (LSM800, Germany). The antibodies used in this study are listed in Table S2.

### RNA extraction and qRT‒PCR

The RNA extraction and qRT‒PCR processes were described in our previous study 21. Gene expression was calculated using the 2^-ΔΔCT^ method and normalized to the internal reference GAPDH as the control group. The qRT‒PCR primer sequences used in this study are listed in Table S3. The qRT‒PCR assay was repeated at least three times.

### *In vivo* mouse models

The protocol for animal experiments was approved by the Animal Management Committee of Wuhan Youdu Biotechnology Co., Ltd. All female nude mice (4-6 weeks of age) were purchased from Beijing Huafukang Biological Polytron Technologies, Inc. and raised in the Experimental Animal Center of Youdu Biotechnology Co., Ltd. For the subcutaneous xenograft tumor model, twelve nude mice were randomly divided into three groups and injected with either stably transduced TIE1-overexpressing or control HeLa cells (5 × 10^6^ cells/0.2 mL in PBS) into the shoulders. Tumor growth was measured every 5 days and the volume of the tumor was calculated with the following formula: length × width^2^ × 0.52 22. When the xenograft tumor diameter reached approximately 0.5 cm, AC-73 (25 mg/kg, #HY-122214, MCE) was injected intratumorally into 4 nude mice with TIE1 overexpression every three days; others were given DMSO intratumoral injection as control. All mice were sacrificed on Day 30 after injection. After sacrifice, tumors were removed for size and weight measurement and embedded in paraffin for IHC staining. For the lung metastasis model, 2 × 10^6^ cells suspended in 0.2 mL PBS were slowly injected into the tail veins. After 6 weeks, the mice were sacrificed, and the lungs were removed and fixed with 4% paraformaldehyde for haematoxylin and eosin (H&E) staining.

### Statistical analysis

Data are presented as the mean ± standard deviation (SD). A value of *P* < 0.05 was considered significant. The difference between two groups was compared by two-sided Student's *t* test. Spearman's test was used to analyze the correlations between two groups. The difference among multiple groups was determined by one-way ANOVA. The Kaplan‒Meier method and log-rank test were used for overall survival (OS) and progression-free survival (PFS) analysis. Univariate and multivariate Cox regression analyses were performed to evaluate independent risk factors for cervical cancer. The χ^2^ test and Fisher's exact test were used to analyze the relationship between TIE1 expression and clinicopathological characteristics. Data were analyzed and plotted using SPSS 20.0 and GraphPad Prism 9.0 software.

## Results

### TIE1 expression correlates with a poor prognosis in cervical cancer patients

**Figure 1:**
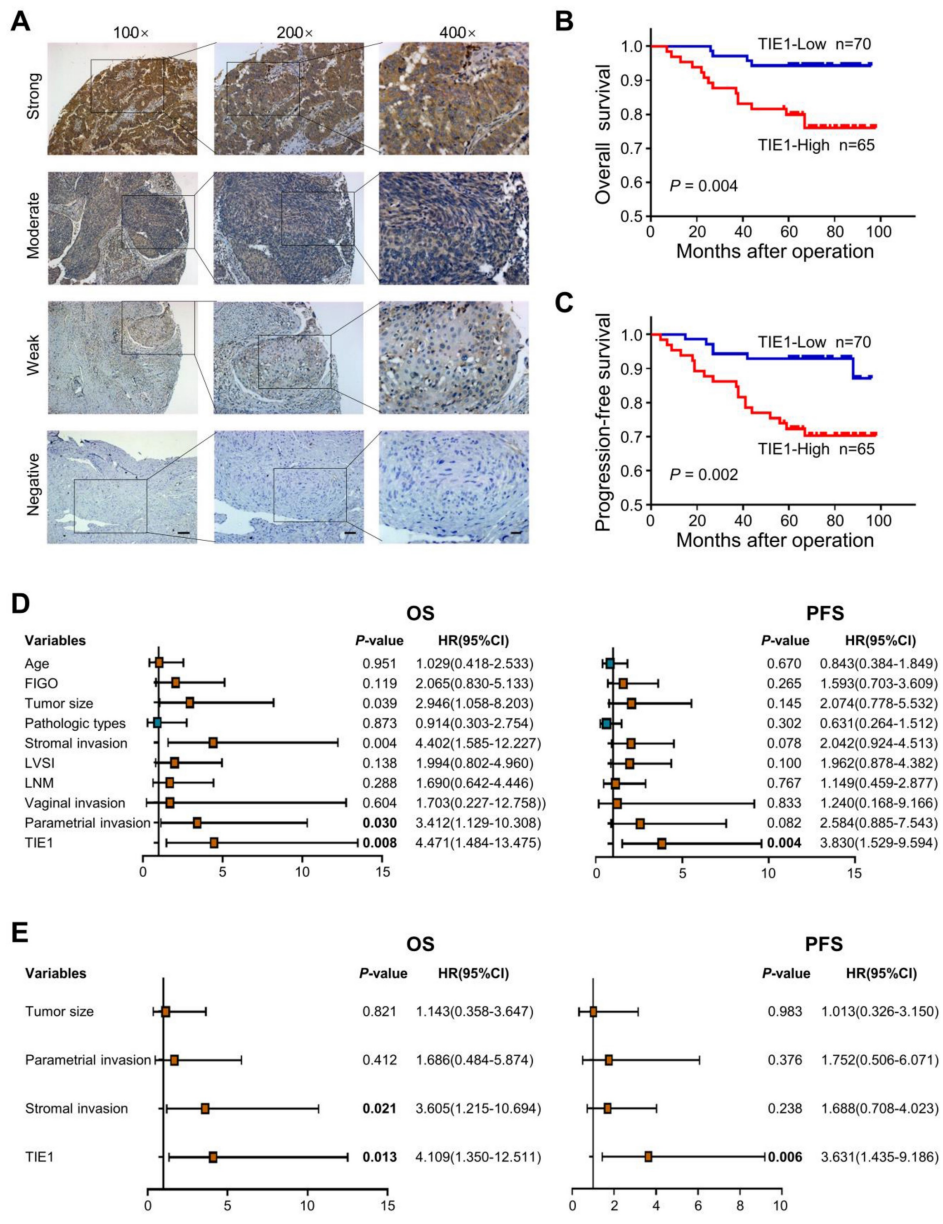
** High expression of TIE1 correlates with a poor prognosis in cervical cancer patients. (A)** Representative IHC images with different TIE1 staining intensities in cervical cancer tissue microarray. Scale bars = 100 μm, 50 μm or 20 μm. **(B-C)** Kaplan‒Meier curves for overall survival (OS) and progression-free survival (PFS) based on TIE1 expression in 135 cervical cancer patients. **(D-E)** Forest plot of univariate and multivariate Cox regression analysis of OS and PFS in 135 cervical cancer patients.

In our previous study, TIE1 was detected by IHC in 80 cervical cancer tissues and 19 normal cervix tissues. Compared to that in normal cervical epithelium, TIE1 expression was higher in cervical cancer epithelial cells 7. To further explore the association between clinicopathological factors and TIE1 expression, we examined the level of TIE1 in 135 cervical cancer patients by IHC. Representative IHC images with different staining intensities were shown in Figure 1A. The clinicopathological analysis indicated that TIE1 expression was positively correlated with lymphovascular space invasion (*P* = 0.010) and lymph node metastasis (*P* = 0.002) (Table 1). Survival analysis showed that patients with high TIE1 expression had significantly shorter OS and PFS than those with low TIE1 expression (*P* = 0.004 and *P =* 0.002, respectively) (Figure 1B-C). We also performed univariate and multivariate Cox regression analysis to assess the prognostic value of TIE1 in cervical cancer (Figure 1D-E). Taken together, these findings indicate that TIE1 is an independent prognostic factor for both OS and PFS in cervical cancer patients.

### TIE1 promotes cervical cancer progression *in vitro* and *in vivo*

**Figure 2:**
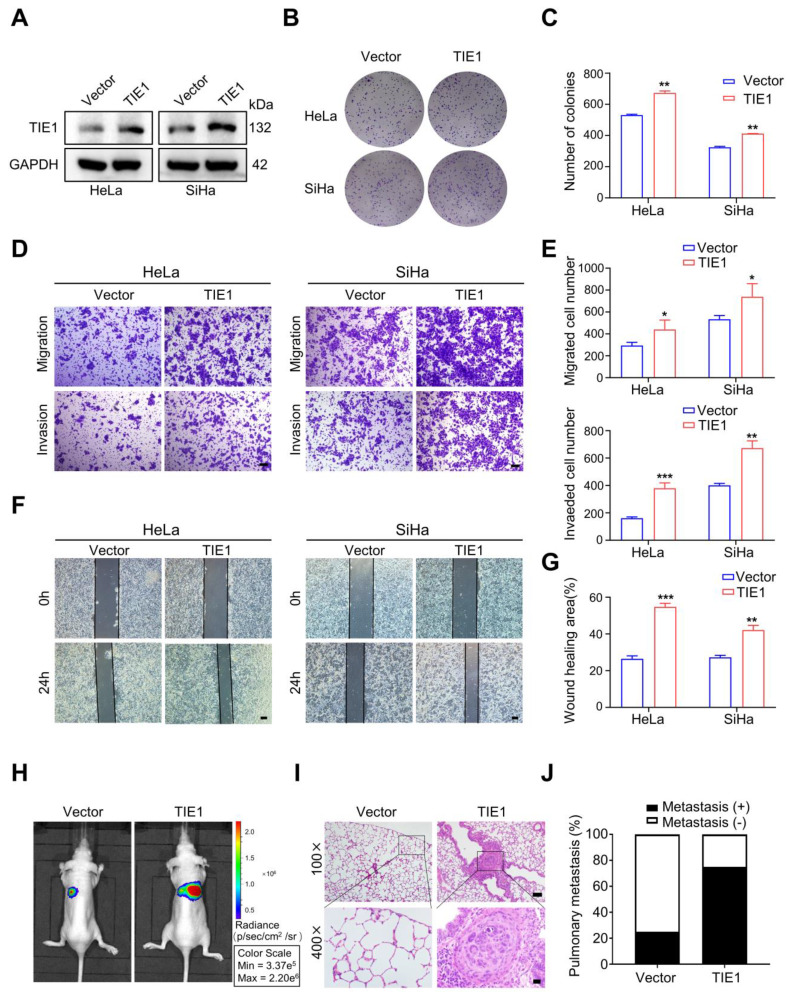
** TIE1 promotes cervical cancer progression *in vitro* and *in vivo*. (A)** The overexpression efficiency of TIE1 was verified by Western blotting assays in HeLa and SiHa cells. **(B-C)** Colony formation experiments confirmed the effects of TIE1 overexpression on cell proliferation in HeLa and SiHa cells. **(D-E)** The effects of TIE1 overexpression on cell migratory and invasive capacities were measured by transwell assays in HeLa and SiHa cells. Scale bars = 50 μm. **(F-G)** The effects of TIE1 overexpression on cell migratory abilities were identified by wound healing assays in HeLa and SiHa cells. Scale bars = 200 μm. **(H-J)** Representative bioluminescence images, H&E staining of pulmonary metastatic foci and the rates of lung metastasis in the control groups and TIE1-overexpressing groups (n = 4 per group). Scale bars = 100 μm and 20 μm. *P < 0.05; **P < 0.01; ***P < 0.001.

To investigate the role of TIE1 in cervical cancer progression, we demonstrated TIE1 was significantly overexpressed in cervical cancer cell lines compared to normal cervical cell line H8 using Western blotting (Figure S1A-B). Furthermore, we used a lentiviral vector to construct SiHa and HeLa cervical cancer cell lines that stably overexpressed FLAG-tagged TIE1. Western blotting was used to verify the overexpression efficiency of TIE1 at the protein level (Figure 2A and Figure S1C). The results from colony formation assays indicated that TIE1 overexpression increased the proliferation of HeLa and SiHa cells (Figure 2B-C). Transwell assays showed that overexpression of TIE1 in HeLa and SiHa cells enhanced cell migratory and invasive abilities compared with those in the vector group (Figure 2D-E). Consistently, wound healing assays showed that TIE1 overexpression notably enhanced the migration of HeLa and SiHa cells (Figure 2F-G). Furthermore, *in vivo* imaging and HE staining of a tail vein injection model showed that the incidence of pulmonary metastasis was increased in the TIE1 overexpression group (Figure 2H-J). In contrast, knockdown of TIE1 in SiHa and HeLa cells significantly weakened cell migratory and invasive capacities (Figure S2). Collectively, these data revealed that TIE1 promotes cervical cancer progression *in vitro* and* in vivo*.

### TIE1 physically interacts with Basigin

**Figure 3:**
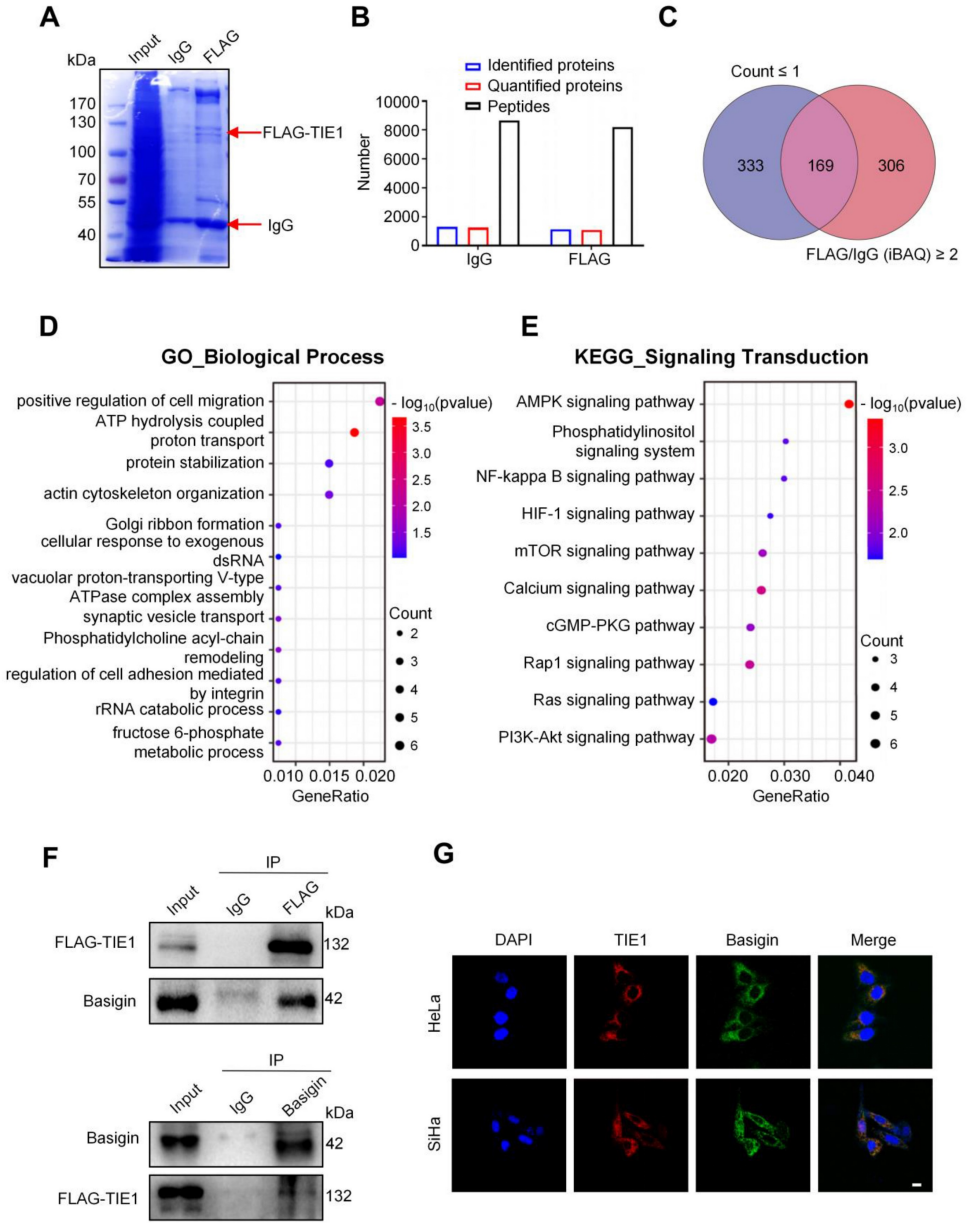
** TIE1 physically associates with Basigin. (A)** Coomassie brilliant blue staining was performed after gel electrophoresis in TIE1-overexpressing HeLa cells using an anti-FLAG antibody for immunoprecipitation. **(B)** The amounts of peptides, identified proteins and quantified proteins of IgG and FLAG groups in the mass spectrometry analysis. **(C)** Venn diagram showing 169 overlapping proteins related to two filters. **(D-E)** GO_BP (Gene Ontology Biological Process) and KEGG (Kyoto Encyclopedia of Genes and Genomes) enrichment analysis based on 169 TIE1-interacting proteins identified by LC‒MS/MS. **(F)** A co-immunoprecipitation (Co-IP) assay was performed in HeLa cells that stably overexpressed FLAG-tagged TIE1; IgG was used as a control. **(G)** Immunofluorescence staining assay was performed in the HeLa and SiHa cell lines. Scale bars = 10 μm.

Protein‒protein interactions play an important role in regulating its function. To explore the mechanism of action of TIE1 in cervical cancer progression, we examined the potential TIE1-interacting proteins by immunoprecipitation (IP) and liquid chromatography coupled with tandem mass spectrometry (LC‒MS/MS) analysis in the TIE1-overexpressing HeLa cell line (Figure 3A). A total of 1128 proteins were identified by mass spectrometry in the FLAG group (Figure 3B). According to the two filters described in the *Materials and methods* section, we screened 169 candidate proteins (Figure 3C), the top 30 of which are listed in Table S1. GO and KEGG analysis indicated that these TIE1-binding proteins were significantly enriched not only in various critical biological processes, such as positive regulation of cell migration, energy metabolism and protein degradation, but also in multiple tumor-associated signalling pathways, among which MAPK signalling ranked first (Figure 3D-E and Figure S3A-B). Additionally, KEGG Enrichment Human Disease indicated that these TIE1-binding proteins were significantly enriched in human papillomavirus infection (Figure S3C), which was closely related with cervical cancer. Furthermore, in the KEGG Enrichment Cellular Process analysis, crucial signalling transduction pathways, including adherens junction and focal adhesion, were enriched (Figure S3D). These enrichments highlighted their relevance in cervical cancer progression.

As TIE1 is mainly located on the cell membrane, we focused on the membrane proteins of these binding candidates. Among them, we chose Basigin for further study because it ranked highly in MS quantitation and has a widely known tumor-promoting role. Next, we validated the binding between TIE1 and Basigin in the TIE1-overexpressing HeLa cell line via Co-IP (Figure 3F). In addition, immunofluorescence staining showed the colocalization of TIE1 and Basigin in HeLa and SiHa cell lines (Figure 3G). In summary, TIE1 could physically interact with Basigin.

### TIE1 increases the expression of Basigin and MMPs

**Figure 4:**
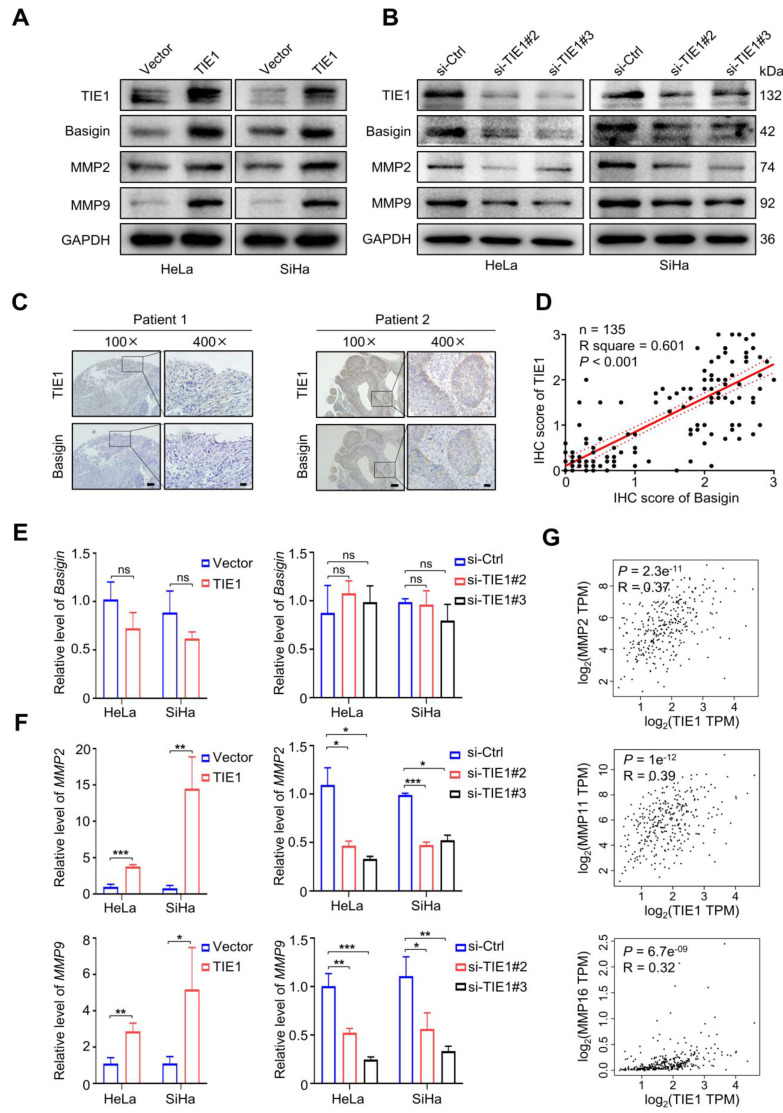
** TIE1 increases the expression of Basigin/MMPs. (A-B)** TIE1, Basigin, MMP2 and MMP9 expression levels was shown by Western blotting in TIE1-upregulated and TIE1-downregulated cells. **(C)** Representative IHC staining of cervical cancer tissue for TIE1 and Basigin expression. Scale bars = 100 μm or 20 μm, respectively. **(D)** Correlations between TIE1 and Basigin expression levels in 135 cervical cancer patients. The *P* value was calculated by the Pearson chi-squared test. **(E-F)** The mRNA expression of Basigin, MMP2 and MMP9 was measured by qRT‒PCR in TIE1-upregulated and TIE1-downregulated cells. **(G)** The correlation between TIE1 and MMPs mRNA levels was analyzed by the GEPIA network tool. *P < 0.05; **P < 0.01; ***P < 0.001; ns, no significance.

Since Basigin was recognized as a regulator of MMPs, we intended to investigate whether TIE1 could regulate the expression of Basigin and MMPs. First, Western blotting analysis showed that TIE1 overexpression notably increased the protein levels of Basigin, MMP2 and MMP9 in HeLa and SiHa cell lines (Figure 4A and Figure S4A-B).

In contrast, knockdown of TIE1 led to a significant decline in the levels of Basigin, MMP2 and MMP9 (Figure 4B and Figure S4C-D). Then, we analyzed the level of Basigin by IHC staining in 135 cervical cancer tissues. The results indicated that there was a positive correlation between Basigin and TIE1 (*P* < 0.001) (Figure 4C-D). Interestingly, we found that overexpression or knockdown of TIE1 had little influence on the expression of Basigin mRNA (Figure 4E) but significantly increased or decreased the mRNA levels of MMP2 and MMP9 (Figure 4F). The GEPIA network analysis tool also showed that TIE1 was positively associated with multiple MMPs at the RNA level (Figure 4G and Figure S5A). Additionally, we observed a positive correlation of TIE1 and MMP2, as well as MMP9 in the GSE9750 dataset (Figure S5B-C).

### TIE1 promotes cervical cancer progression by regulating Basigin

**Figure 5:**
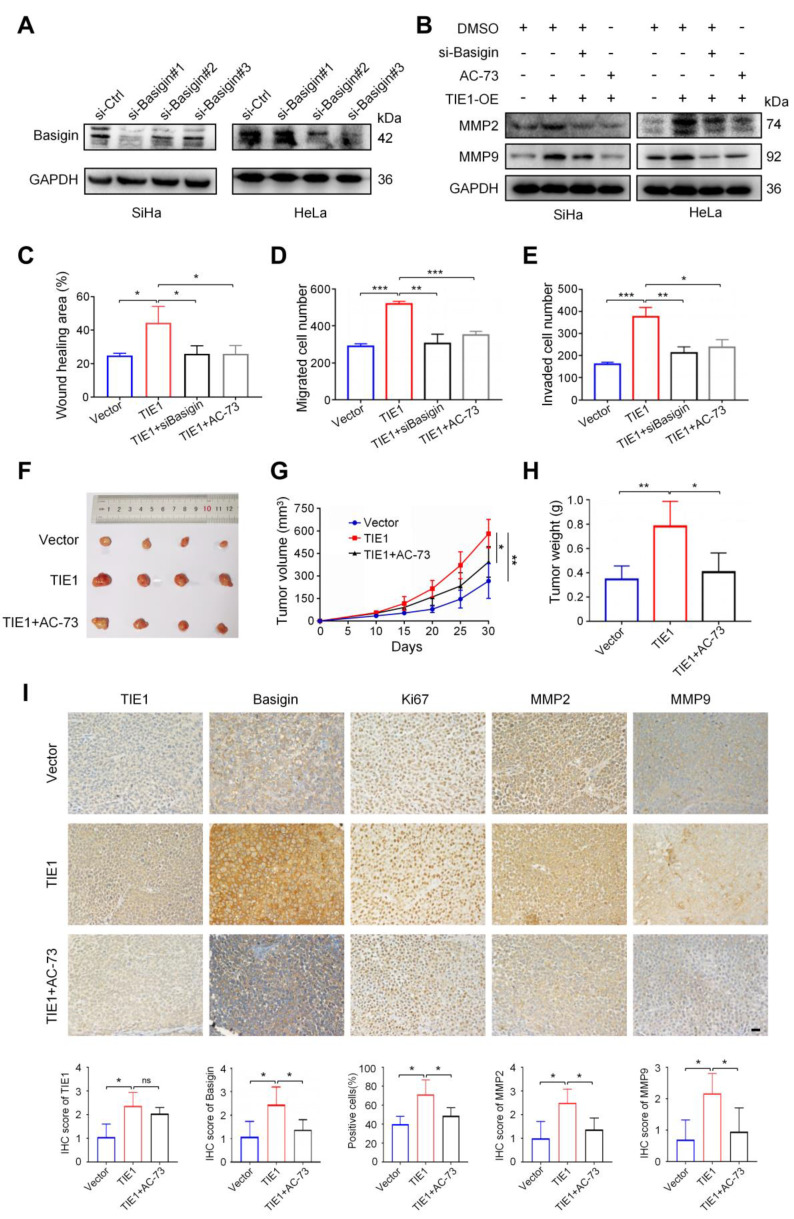
** TIE1 promotes cervical cancer progression via the Basigin/MMPs pathway. (A)** The knockdown efficiency of Basigin was verified by Western blotting assay in HeLa and SiHa cells, and si-Basigin#2 was selected for further study. **(B)** Western blotting showing the levels of MMP2 and MMP9 under the indicated treatments. **(C-D)** Transwell assays were used to investigate the cell migratory and invasive capacities of HeLa-Vector, HeLa-TIE1, HeLa-TIE1 with si-Basigin or AC-73 (Basigin inhibitor). **(E)** Wound healing assays were performed to detect cell migratory abilities in different treatment groups. **(F-H)** Representative pictures of subcutaneous tumors, tumor growth curves and tumor weights in the HeLa-Vector group, HeLa-TIE1 group and HeLa-TIE1-AC73 group (n = 4 per group). **(I)** Representative IHC images and histogram analysis showing the expression of TIE1, Basigin, Ki-67, MMP2, and MMP9 on the same tissue sections in the indicated group. Scale bars = 20 μm. * *P* < 0.05, ** *P* < 0.01, *** *P* < 0.001.

To further verify that TIE1 promoted cervical cancer progression by regulating Basigin, we applied siRNA or an inhibitor (AC-73) of Basigin to TIE1-overexpressing HeLa and SiHa cell lines. The knockdown efficiency of Basigin was verified by Western blotting (Figure 5A and Figure S4E). Our results indicated that both Basigin siRNA and AC-73 could reverse MMP2 and MMP9 upregulation induced by TIE1 overexpression (Figure 5B and Figure S4F). Wound healing and transwell assays demonstrated that the increased migration and invasion abilities induced by TIE1 overexpression were obviously weakened after Basigin siRNA or AC-73 treatment (Figure 5C-E), with representative pictures shown in Figure S6 and Figure S7. Consistent with the *in vitro* experiments, AC-73 treatment attenuated the increase in xenograft tumor volume and weight induced by TIE1 overexpression (Figure 5F-H). It has been reported that AC-73 could specifically disrupt Basigin dimerization, thereby suppressing the Basigin/ERK1/2/MMPs pathways 23, 24. So, we conducted a Western blotting experiment, confirming that TIE1 can activate MAPK/ERK pathway in cervical cancer cells (Figure S8). In addition, IHC staining of nude mouse xenograft tissues showed that the levels of Ki67, MMP2 and MMP9 were increased in the TIE1 overexpression group but decreased in the group with TIE1 overexpression combined with AC-73 treatment (Figure 5I). Moreover, endothelial cells in xenograft cervical tumor tissues were stained with anti-CD31 and anti-CD105 to assess angiogenesis activity by Microvessel Density (MVD). The results showed a higher MVD in the TIE1 overexpression group. However, AC-73 treatment attenuated the enhanced MVD induced by TIE1 overexpression, suggesting that TIE1 promoted xenograft tumor angiogenesis through Basigin *in vivo* (Figure S9). In summary, Basigin is an essential factor for TIE1-mediated cervical cancer progression.

### TIE1 enhances the stability of Basigin

**Figure 6:**
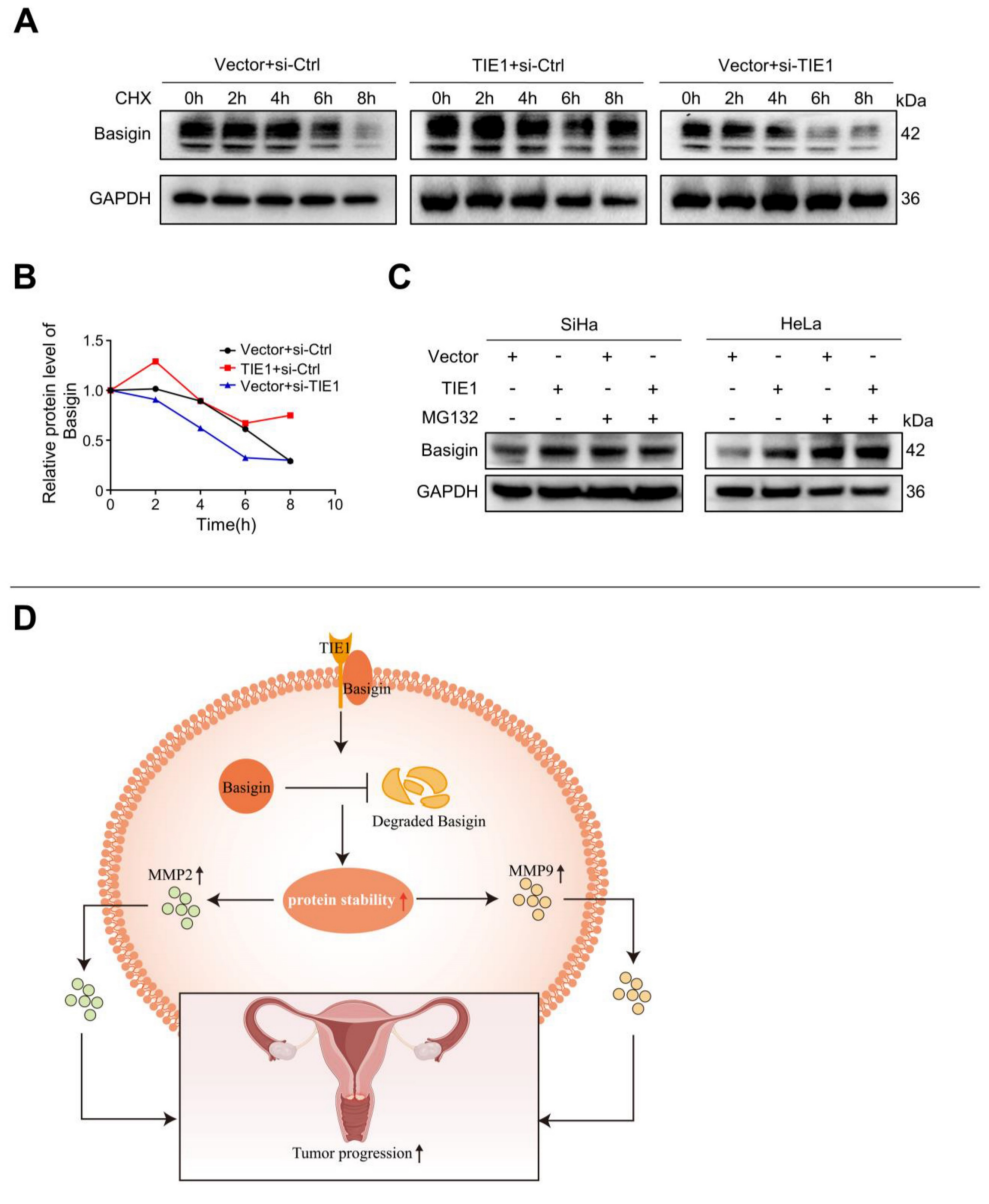
**TIE1 could enhance the stability of Basigin**. **(A-B)** The cycloheximide (CHX) experiment was performed to detect the expression of Basigin at the indicated time points in the TIE1 overexpression or knockdown HeLa cells. **(C)** The expression of Basigin was demonstrated by Western blotting in HeLa cells with TIE1 overexpression or low expression treated with or without MG132. **(D)** Schematic diagram: TIE1 could bind to and stabilize Basigin to promote MMPs expression, resulting in tumor growth and metastasis of cervical cancer.

Since TIE1 regulated Basigin protein expression without obviously affecting its mRNA level, we hypothesized that TIE1 could enhance the stability of Basigin. To test this speculation, we inhibited protein synthesis using cycloheximide (CHX) and observed the degradation process of Basigin in the HeLa cell line. The results indicated that TIE1 overexpression extended the protein half-life of Basigin, while its silencing significantly accelerated the degradation of Basigin proteins (Figure 6A-B). Moreover, TIE1 overexpression hardly changed Basigin protein levels when proteasome-mediated protein degradation was blocked by MG132 treatment (Figure 6C and Figure S4G). In conclusion, TIE1 could stabilize Basigin in cervical cancer.

## Discussion

Receptor tyrosine kinases (RTKs) play important roles in multiple cellular processes, such as cell growth, migration, differentiation and metabolism 25. Abnormal RTKs expression can cause a variety of diseases, especially cancer 26. In cervical cancer, RTKs-targeted therapy, such as VEGF/VEGFR inhibitors, significantly improves the survival prognosis of patients 27, 28. The ANG/TIE signalling pathway contains two highly homologous receptor tyrosine kinases, TIE1 and TIE2. Most previous studies focused on their role in vascular endothelial cells 5, 29, with fewer studies on tumor cells. This study concentrated on the orphan receptor tyrosine kinase TIE1 in cancer cells and explored its function and mechanism of action in cervical cancer.

We found that TIE1 was upregulated in human cervical cancer and correlated with a poor prognosis. Consistent with our results, it has been indicated that elevated TIE1 expression is associated with a poor prognosis in patients with gastric cancer, ovarian cancer and metastatic breast cancer 30-32. Our functional assays demonstrated that TIE1 promoted cervical cancer growth and metastasis. In addition, TIE1 has been shown to promote cisplatin resistance in ovarian cancer by upregulating xeroderma pigmentosum complementation group C (XPC)-mediated nucleotide excision repair (NER), decreasing cell sensitivity to cisplatin 31. Although our study did not explore the relationship between TIE1 and cervical cancer chemotherapy resistance, it prompts consideration of whether TIE1-mediated regulation of DNA damage repair also occurs in cervical cancer.

We identified a batch of possible TIE1-binding proteins by Co-IP/MS. Among them, we selected Basigin for further research and verified its interaction with TIE1. Our experiments demonstrate that TIE1 overexpression promotes Basigin protein stability. The cycloheximide (CHX) experiment revealed a significantly slower degradation rate of Basigin in cells overexpressing TIE1 compared to control cells, suggesting that TIE1 may inhibit Basigin protein degradation. Additionally, treatment with MG132, a proteasome inhibitor, resulted in minimal changes in Basigin protein levels in TIE1-overexpressing cells compared to control cells, indicating that TIE1 may protect Basigin from proteasomal degradation.

In eukaryotic cells, damaged proteins and cellular components are typically cleared through degradation pathways mediated by proteasomes or lysosomes. The ubiquitin-proteasome system is responsible for targeting short-lived and soluble misfolded proteins, while lysosomes degrade long-lived proteins, insoluble aggregates, and organelles 33. Our findings suggest that TIE1 may modulate Basigin protein stability by potentially interfering with its degradation via the ubiquitin-proteasome pathway. Furthermore, we propose that TIE1 may also modulate Basigin protein turnover through post-translational modifications or by influencing protein-protein interactions. we propose that TIE1 may also modulate post-translational modifications of Basigin, or influence protein-protein interactions with Basigin. Future studies employing techniques such as co-immunoprecipitation and ubiquitination assays could provide further insights into the specific mechanisms underlying TIE1-mediated regulation of Basigin protein stability. When detecting TIE1 and Basigin by Western blotting, double bands occasionally appeared, which may be attributed to different degrees of glycosylation 34. Membrane protein glycosylation plays an important role in regulating ligand binding and protein interactions. Some studies have demonstrated that the glycosylation level of Basigin could affect the expression of MMPs 35. Although this project did not investigate the posttranslational modification of TIE1 and Basigin, further research to explore whether TIE1 affects the protein modification of Basigin is still worthwhile. Simultaneously, our study revealed that TIE1-Basigin pathway was participated in angiogenesis, suggesting a potential novel mechanism independent of TIE2. Further research is warranted to provide detailed insights into this discovery.

We demonstrated that TIE1 increases the expression of Basigin and MMPs by Western blot assay. While measuring MMP-2 and MMP-9 activities would undoubtedly provide valuable insights, our current investigation focuses on understanding TIE1's broader impact on the cervical cancer progression. Consequently, we have chosen not to include these measurements in this particular investigation. It's worth noting that *Zhao SH et al.* 36 and *Wu J. et al.* 37 reported Basigin overexpression promoting the MMP-2 and MMP-9 activities using gelatin zymography. However, we fully recognize the significance of MMP-2 and MMP-9 in cervical cancer progression and plan to explore their activity in future investigations.

Considering that Basigin plays a significant role in TIE1-mediated cervical cancer progression, Basigin inhibitors may be effective for cervical cancer patients with high TIE1 expression. Currently, several types of Basigin inhibitors are under clinical trials 38, 39. Although few studies have focused on therapy targeting TIE1, a nanobody with high expression, good affinity, and specificity for TIE1 was recently developed to trigger TIE1-dependent inhibition of TIE2 phosphorylation and angiogenesis in endothelial cells 40. Similarly, novel drugs that disrupt the TIE1-Basigin association could be developed as new agents for cervical cancer treatment.

## Conclusions

In summary, our study provides compelling evidence that high TIE1 expression is clinically and functionally significant in the progression of cervical cancer; TIE1 acts by stabilizing Basigin and increasing MMPs levels. Furthermore, TIE1 has potential as a novel prognostic factor in cervical cancer. Our findings not only provide new insights into the molecular mechanism underlying cervical cancer progression but also provide a new potential therapeutic target for cervical cancer patients.

## Supplementary Material

Supplementary figures and tables; methods.

## Figures and Tables

**Table 1:** Correlation between TIE1 expression and clinicopathologic characteristics of cervical cancer.

Characteristics	Total	TIE1 expression		*P*-value
	135	High	Low	
Age (years)				0.272
< 47	64	34	30	
≥ 47	71	31	40	
FIGO stage				0.647
Ⅰ (Ⅰa2+Ⅰb1+Ⅰb2)	98	46	52	
Ⅱ (Ⅱa1+Ⅱa2)	37	19	18	
Tumor size (cm)				0.048
≤ 4	118	53	65	
> 4	17	12	5	
Pathologic types				0.821
Squamous cell carcinoma	109	53	56	
Adenocarcinoma	26	12	14	
Differentiation				0.937
Well	17	8	9	
Moderate	81	40	41	
Poor	37	17	20	
Stromal invasion				0.476
< 1/2	79	36	43	
≥ 1/2	56	29	27	
LVSI				0.010
Positive	38	25	13	
Negative	97	40	57	
LNM				0.002
Positive	30	22	8	
Negative	105	43	62	
Vaginal invasion				0.352
Positive	4	3	1	
Negative	131	62	69	
Parametrial invasion				0.658
Positive	11	6	5	
Negative	124	59	65	

Χ^2^-test. *FIGO* the International Federation of Gynecology and Obstetrics,* LVSI* lymphovascular space invasion; *LNM* lymph node metastasis.

## Abbreviations

TIE1: tyrosine kinase with immunoglobulin and EGF-like domains 1
MMPs: matrix metalloproteinases
Co-IP/MS: co-immunoprecipitation and mass spectrometry
LC‒MS/MS: liquid chromatography coupled with tandem mass spectrometry
MVD: Microvessel Density
GO: Gene Ontology
GO_BP: Gene Ontology Biological Process
GO_CC: Gene Ontology Cellular Component
GO_MF: Gene Ontology Molecular Function
KEGG: Kyoto Encyclopedia of Genes and Genomes
CHX: cycloheximide
GEPIA: Gene Expression Profiling Interactive Analysis
GAPDH: glyceraldehyde-3 phosphate dehydrogenase
FIGO: International Federation of Gynecology and Obstetrics
LVSI: lymphovascular space invasion
LNM: lymph node metastasis
