# Supplementary material for: TIE1 promotes cervical cancer progression via Basigin-matrix metalloproteinase axis
Source: Int J Biol Sci. 2024 Apr 8;20(6):2297–309. doi: 10.7150/ijbs.93667 (PMC11008262; doi:10.7150/ijbs.93667)
Supplement: Supplementary file 1 — Supplementary figures and tables; methods. [file ijbsv20p2297s1.pdf]

Figure S1

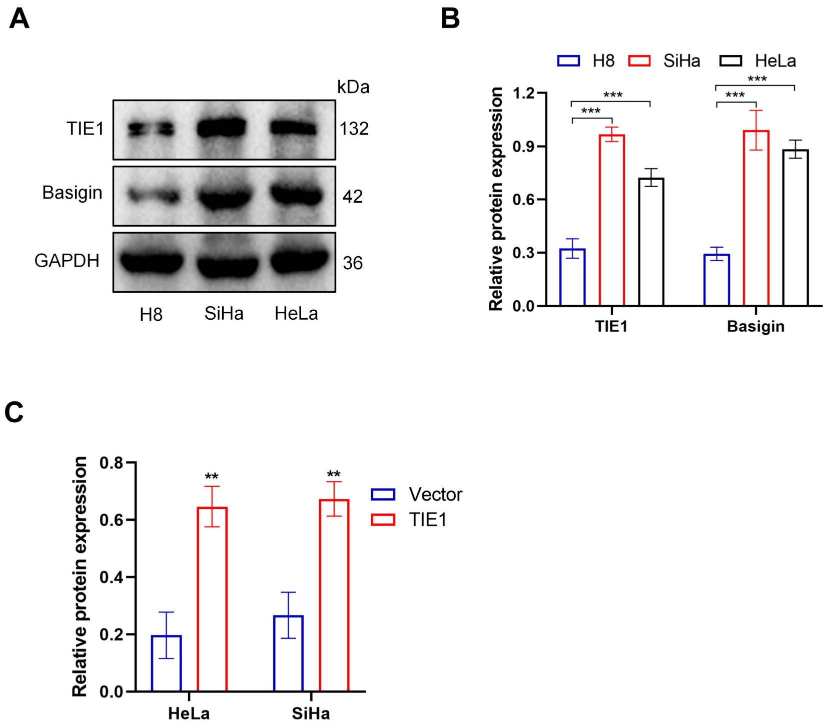

Figure S1. (A-B) Illustrating he expression of TIE1 and Basigin in the normal cervical cell line H8 and cervical cancer cell lines SiHa and HeLa by Western blot analysis. (C) Histogram illustrating the efficiency of TIE1 overexpression. (* $P < 0.05$, ** $P < 0.01$, *** $P < 0.001$)

Figure S2

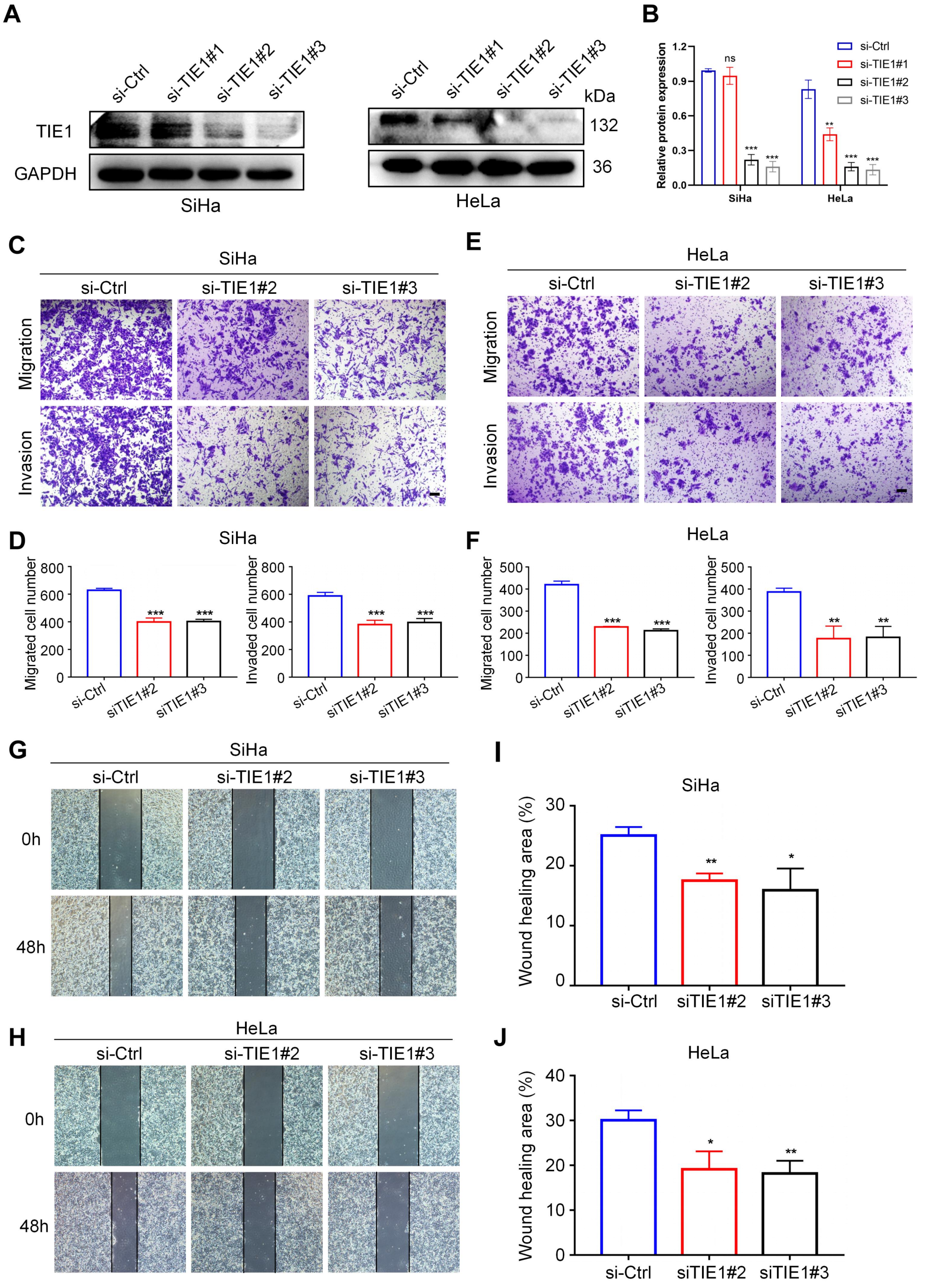

Figure S2. TIE1 knockdown inhibits the migration and invasion of cervical cancer cells. (A-B) The knockdown efficiency of TIE1 was verified by western blotting assays in HeLa and SiHa cells. (C-F) The effects of TIE1 knockdown on cell migratory and invasive capacities were examined by transwell assays in HeLa and SiHa cells. Scale bars = 50 μm. (G-J) The effects of TIE1 knockdown on cell migratory abilities were detected by wound healing assays in HeLa and SiHa cells. Scale bars = 200 μm. (* $P < 0.05$, ** $P < 0.01$, *** $P < 0.001$; ns, no significance)

Figure S3

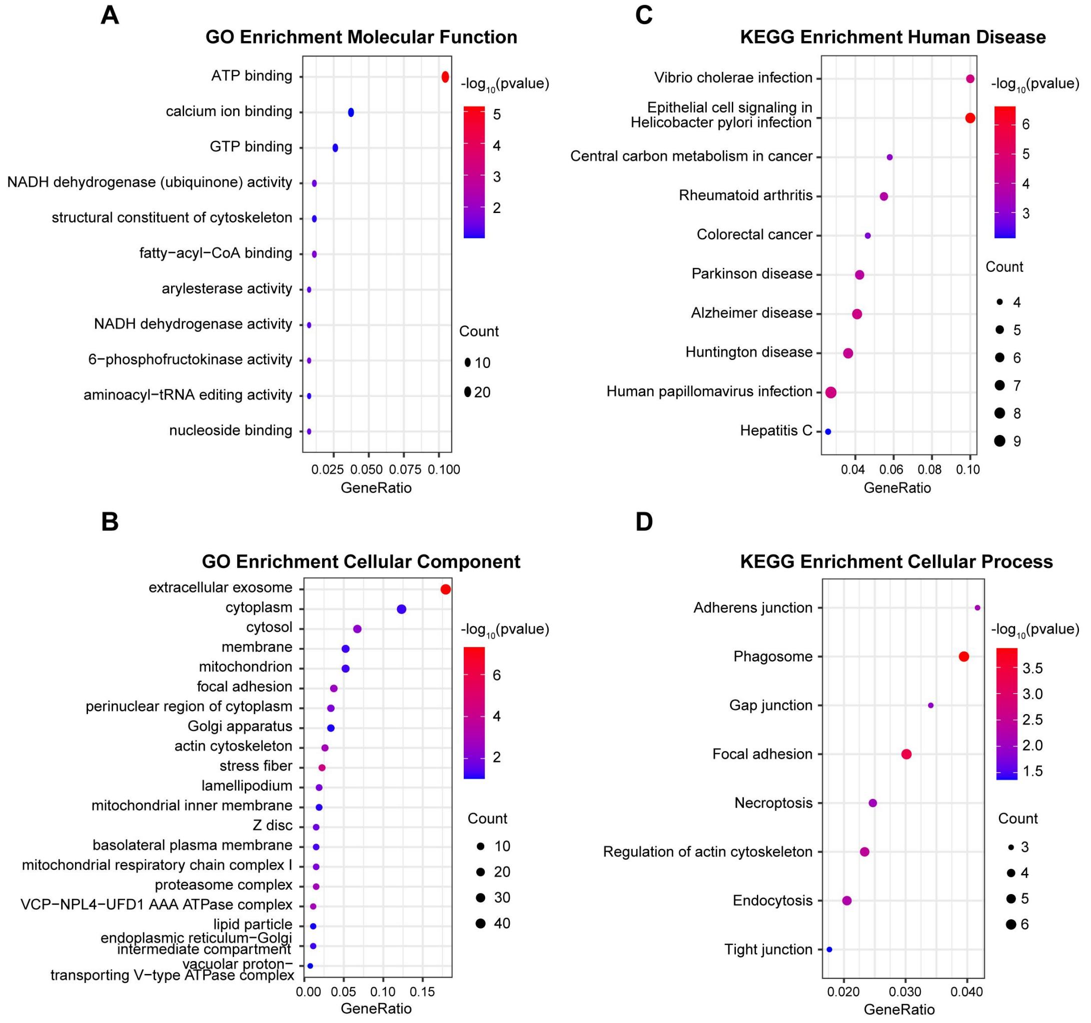

Figure S3. GO and KEGG enrichment analysis based on 169 TIE1-interacting proteins identified by LC–MS/MS. (A-B) GO_MF (Gene Ontology Cellular Component) and GO_CC (Gene Ontology Molecular Function) enrichment analysis based on 169 TIE1-interacting proteins identified by LC–MS/MS (liquid chromatography coupled with tandem mass spectrometry). (C-D) KEGG (Kyoto Encyclopedia of Genes and Genomes) enrichment analysis of different diseases and cellular processes based on 169 TIE1-interacting proteins identified by LC–MS/MS.

Figure S4

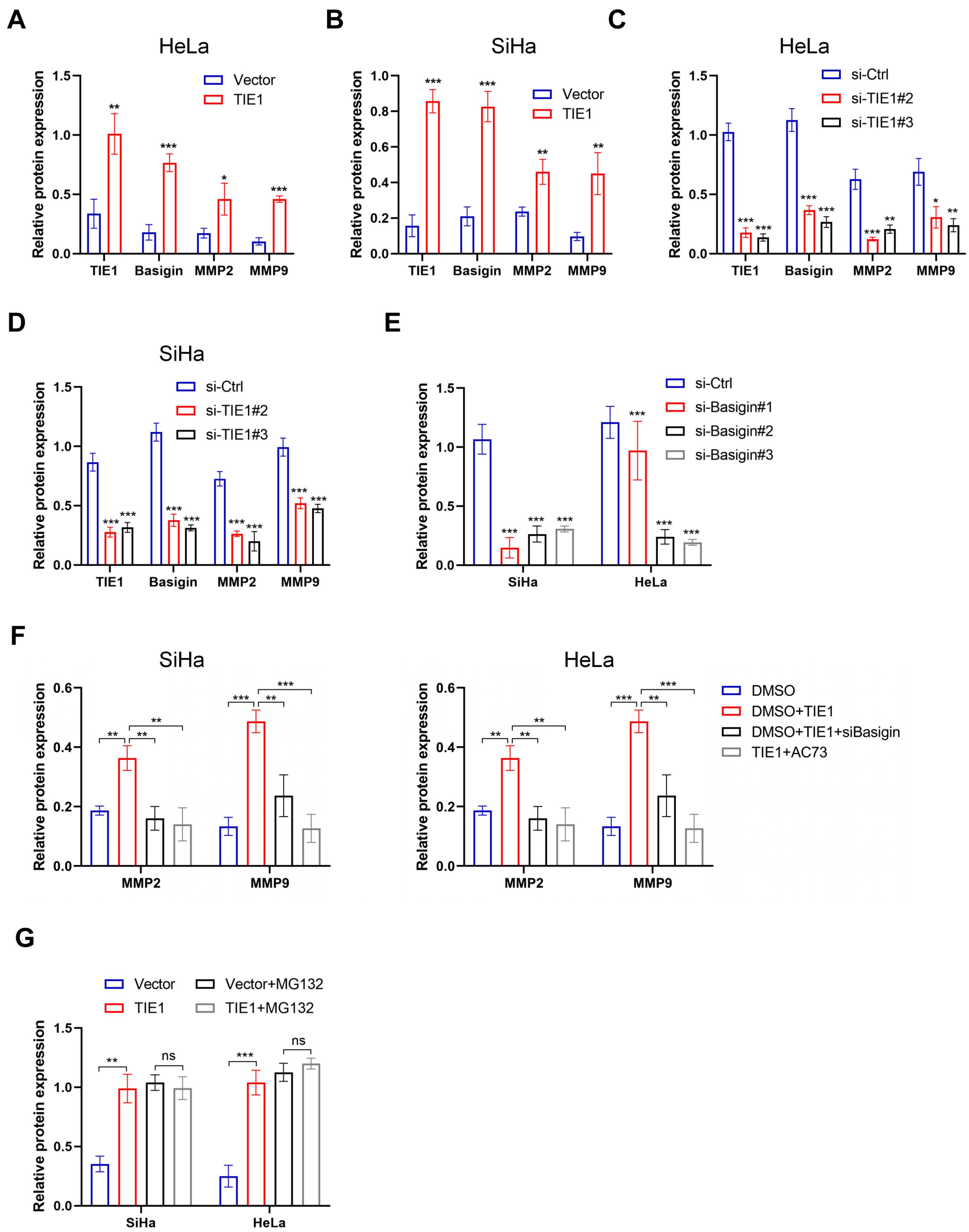

Figure S4. Quantification of Western blotting in Figure 4A (A-B), Figure 4B (C-D), Figure 5A (E), Figure 5B (F), and Figure 6C (G) of the manuscript. (* $P < 0.05$, ** $P < 0.01$, *** $P < 0.001$; ns, no significance).

Figure S5

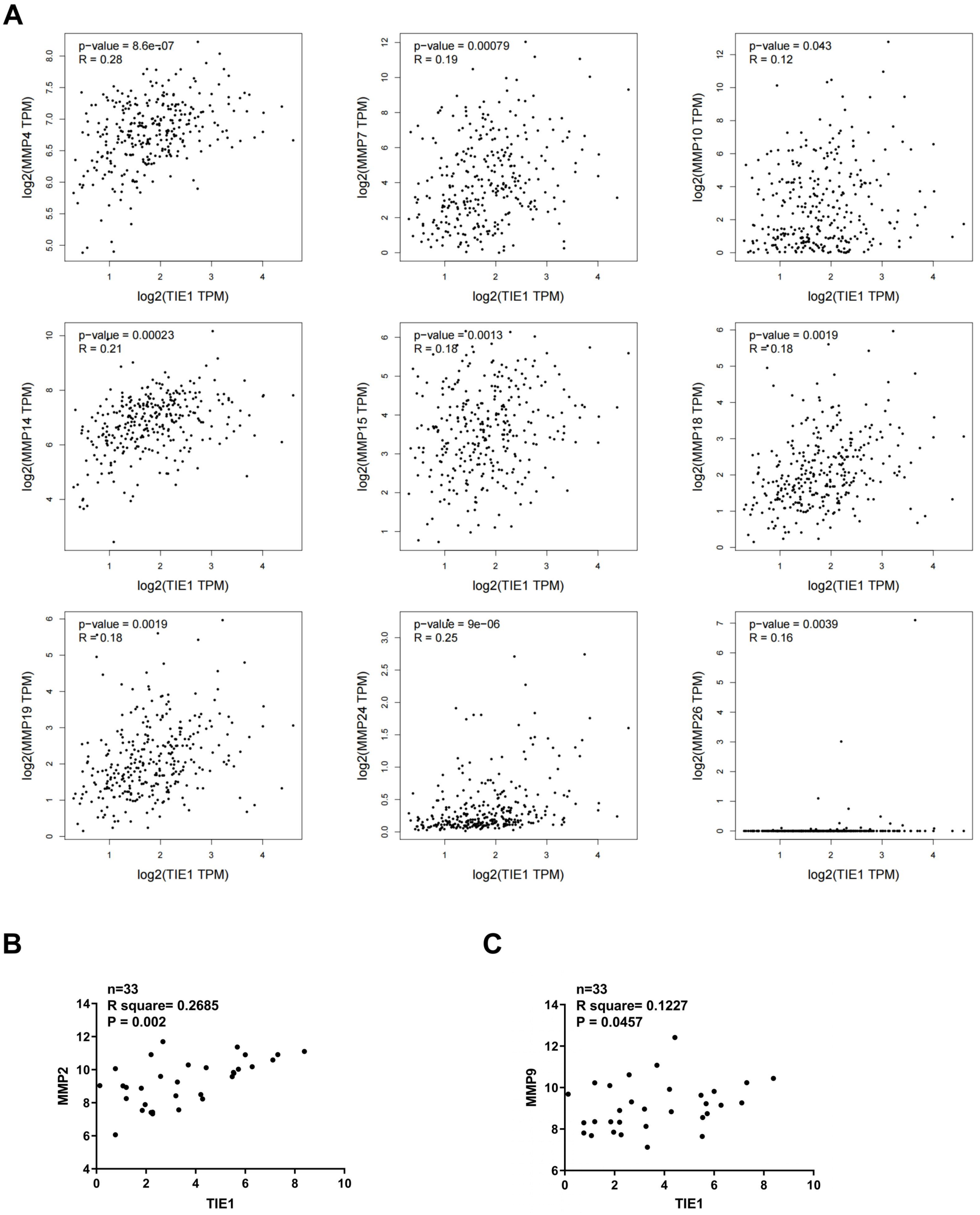

Figure S5. TIE1 is positively correlated with CD147. (A) The correlation between TIE1 and MMPs mRNA levels analyzed by the GEPIA (Gene Expression Profiling Interactive Analysis) network tool. (B-C) The correlation between TIE1 and MMP2, as well as MMP9 mRNA levels in the GSE9750 dataset.

Figure S6

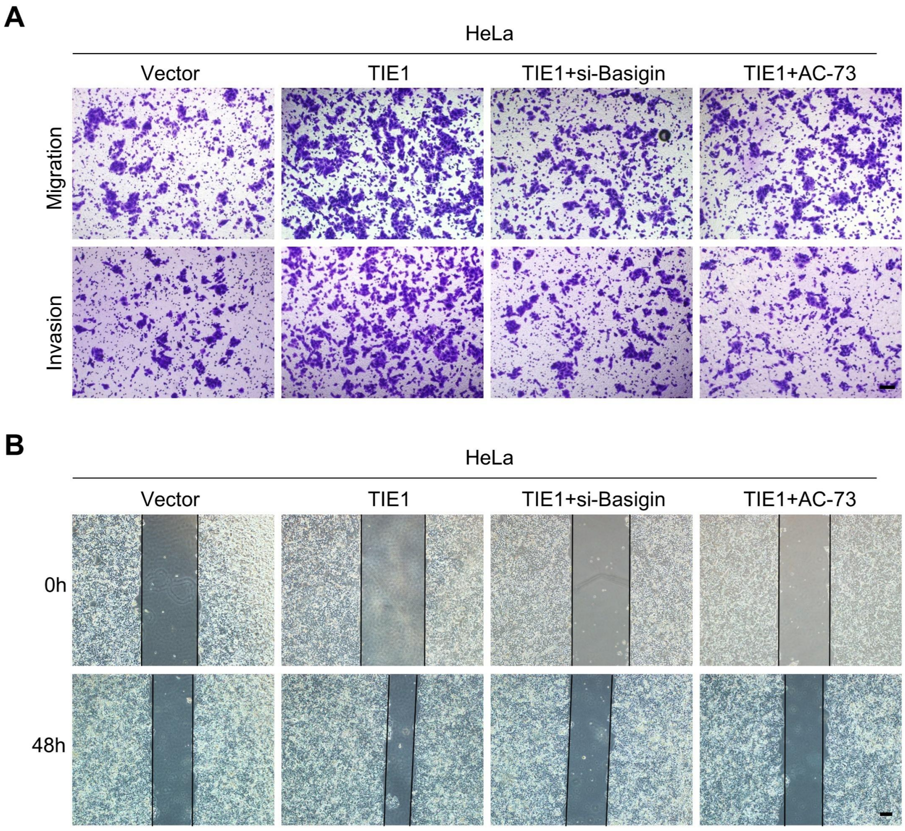

Figure S6. TIE1 promotes invasion and migration of HeLa cervical cancer cell via the Basigin/MMPs pathway. (A) Transwell assays were used to investigate the cell migratory and invasive capacities in HeLa-Vector, HeLa-TIE1, HeLa-TIE1 with si-Basigin or AC-73 cells. Scale bars = 50 μm. (B) Wound healing assays were performed to detect cell migratory abilities in the indicated groups. Scale bars = 200 μm.

Figure S7

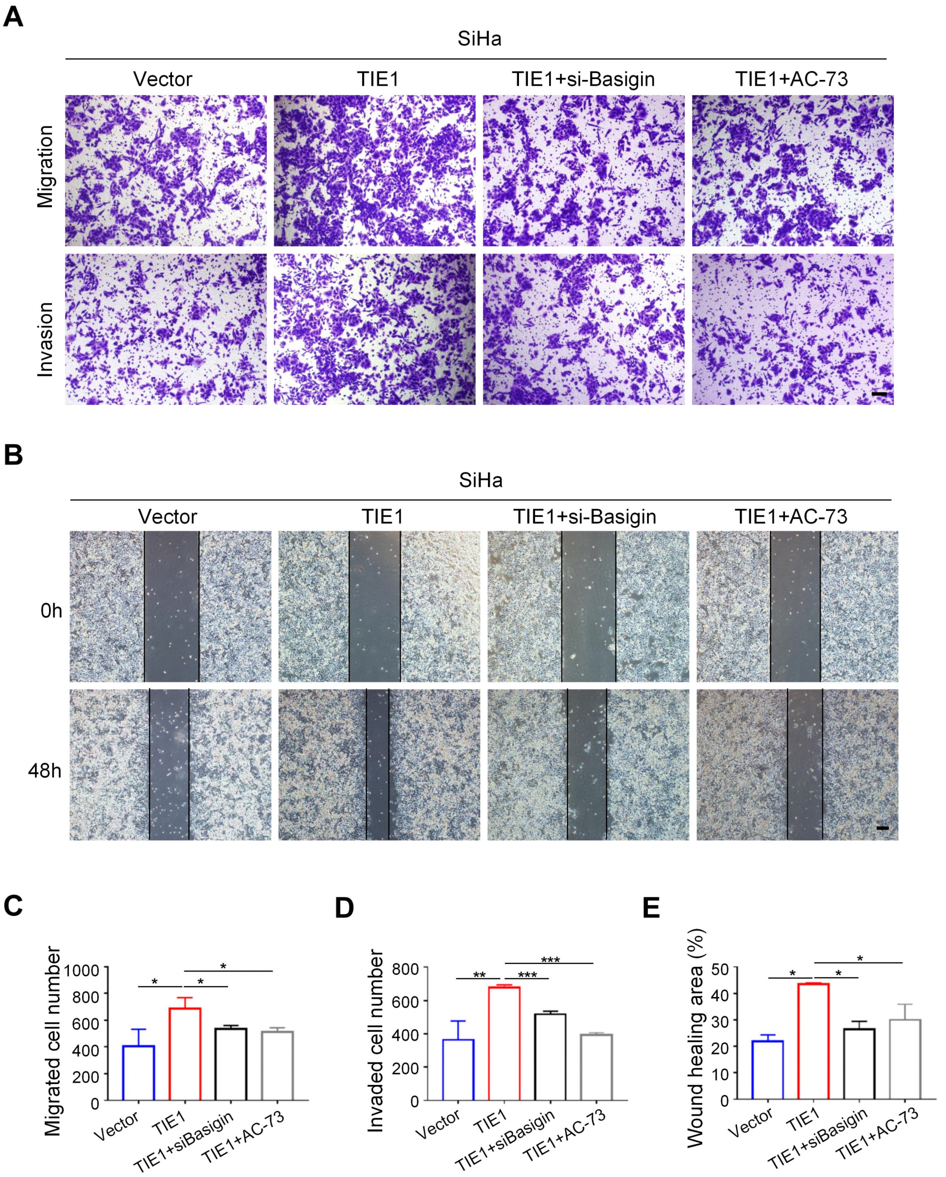

Figure S7. TIE1 promotes invasion and migration of SiHa cervical cancer cell via the Basigin/MMPs pathway. (A) Transwell assays were used to investigate the cell migratory and invasive capacities of SiHa-Vector, SiHa-TIE1, SiHa-TIE1 with si-Basigin or AC-73. Scale bars = 50 μm. (B) Wound healing assays were performed to detect cell migratory abilities in the indicated groups. Scale bars = 200 μm. (C-D) Histogram of transwell assays in the indicated groups. (E) Histogram of wound healing assays in the indicated groups. (* $P < 0.05$, ** $P < 0.01$, *** $P < 0.001$)

Figure S8

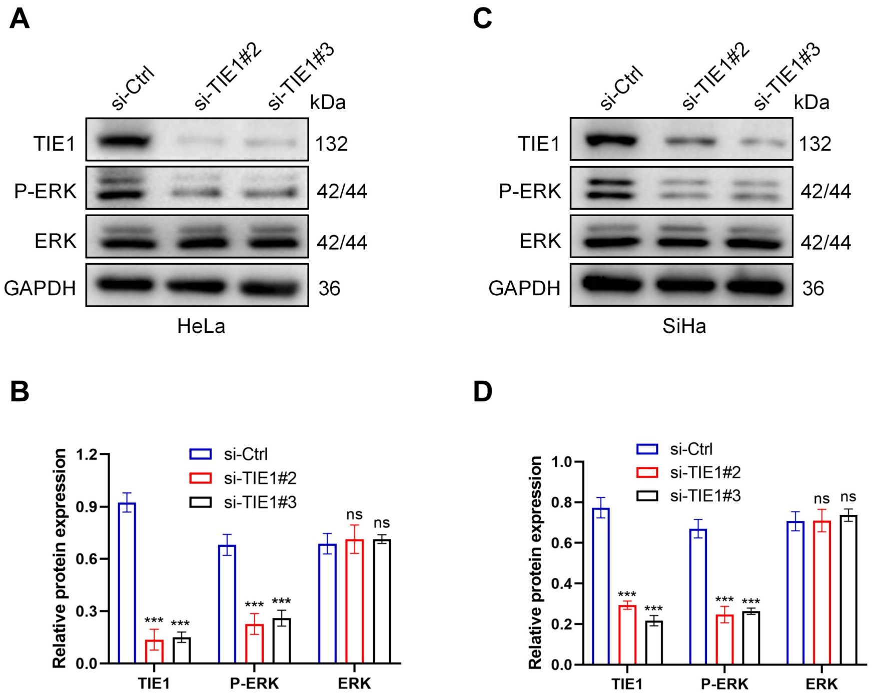

Figure S8. TIE1 activates MAPK/ERK pathway in the cervical cancer cells. (A-D): TIE1, P-ERK and ERK expression levels was shown by Western blotting in TIE1-downregulated cells. (*** $P < 0.001$; ns, no significance).

Figure S9

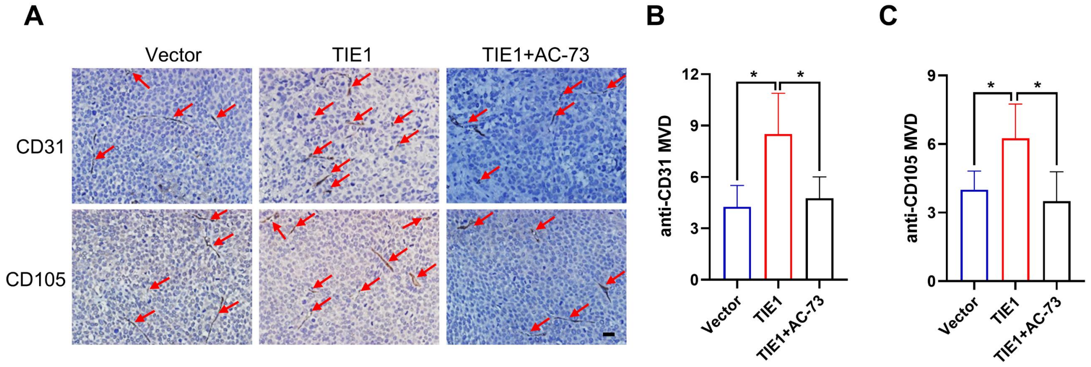

Figure S9. TIE1 promotes xenograft tumor angiogenesis through Basigin. (A) Representative IHC images with different CD31 and CD105 staining intensities in subcutaneous xenografts of nude mice. Scale bars = 20 μm. (B-C) Histograms of MVD (Microvessel Density) showing the results of IHC staining with anti-CD31 (B) and anti-CD105 (C). * $P < 0.05$

Table S1 Top 30 proteins related to TIE1 identified by LC–MS/MS

Gene	Description	FLAG/IgG (iBAQ)	Subcellular Location
DNAJA2	DnaJ homolog subfamily A member 2 (Fragment)	471.0055	Membrane
HIST1H2AJ	Histone H2A type 1-J	370.0304	Nucleus
TOMM5	Mitochondrial import receptor subunit TOM5 homolog	351.6996	Mitochondrion
NDUFB4	NADH dehydrogenase [ubiquinone] 1 beta subcomplex subunit 4	301.6852	Mitochondrion
EFNA1	Ephrin-A1	299.8922	Membrane
SCD5	Stearoyl-CoA desaturase 5	275.193	Endoplasmic reticulum membrane
KIF11	Kinesin-like protein KIF11	269.8848	Cytoplasm
DCUN1D5	DCN1-like protein 5	235.9602	Cytoplasm/ Nucleus
ATP6V0D1	V-type proton ATPase subunit d 1 (Fragment)	228.7614	Whole cell
TMED9	Transmembrane emp24 domain-containing protein 9	197.1568	Endoplasmic reticulum membrane
CDA	Cytidine deaminase	192.0449	Cytosol
PRPS1	Phosphoribosylpyrophosphate synthetase isoform (Fragment)	I176.3136	Cytoplasm
PGAM5	Serine/threonine-protein phosphatase PGAM5	159.1893	Mitochondrion
ATP5PD	ATP synthase subunit d, mitochondrial	102.8288	Mitochondrion
PSME3	Proteasome activator complex subunit 3	96.8111	Nucleus/ Cytoplasm
BSG	Basigin	96.4962	Membrane
TRIM29	Tripartite motif-containing protein 29	95.6173	Cytoplasm

SDF4	45 kDa calcium-binding protein (Fragment)	94.092	Membrane
PSMA5	Proteasome subunit alpha type-5	87.8335	Nucleus/ Cytoplasm
CDC37	Hsp90 co-chaperone Cdc37 (Fragment)	78.9025	Cytoplasm
TMSB4X	Thymosin beta-4	75.1135	Cytoplasm
LIN7A	Protein lin-7 homolog A (Fragment)	74.7033	Membrane
CORO1B	Coronin-1B	71.904	Cytoplasm
STMN2	Stathmin-2	68.994	Cell projection
ATP6V0A1	V-type proton ATPase subunit a 1	66.4041	Cytoplasmic Vesicle
DSTN	Destrin	65.8358	Cytoskeleton
ALPG	Alkaline phosphatase, germ cell type	62.7655	Membrane
TIE1	Tyrosine-protein kinase receptor Tie-1	59.4086	Membrane
CD58	Lymphocyte function-associated antigen 3 (Fragment)	58.7045	Membrane
STK25	Serine/threonine-protein kinase 25 (Fragment)	58.3435	Cytoplasm
SLC25A44	Solute carrier family 25 member 44	53.3975	Mitochondrion

64

Table S2 Antibodies used in the study

Antibody	Catalog#	Working concentration	Manufacturer
TIE1	sc-365961	1:50	Santa Cruz
FLAG	20543-1-AP	1:5000	Proteintech
BSG	ab188190	1:1000 (WB)<br>1:200 (IF)	Abcam
BSG	11989-1-AP	1:1000	Proteintech
GAPDH	10494-1-AP	1:5000	Proteintech
MMP2	ab86607	1μg/mL (WB)<br>5μg/mL (IHC)	Abcam
MMP9	13667	1:1000 (WB)<br>1:200 (IHC)	CST
Ki-67	23709-1-AP	1:6000	Proteintech

Table S3 Primers for qRT–PCR and sequences of siRNAs

TIE1 primer	Forward: 5′-GTGAACAAAGGTGACACCGC-3′
	Reverse: 5′-ACTGTAGATGCCGCTCGATG-3′
Basigin primer	Forward: 5′-GAAGTCGTCAGAACACATCAACG-3′
	Reverse: 5′-TTCCGGCGCTTCTCGTAGA-3′
MMP2 primer	Forward: 5′-TACAGGATCATTGGCTACACACC-3′
	Reverse: 5′-GGTCACATCGCTCCAGACT-3′
MMP9 primer	Forward:5′- TGTACCGCTATGGTTACACTCG-3′
	Reverse: 5′- GGCAGGGACAGTTGCTTCT-3′
GAPDH primer	Forward: 5′- ATCACCATCTTCCAGGAGCGA-3′
	Reverse: 5′- CCTTCTCCATGGTGGTGAAGAC-3′
siTIE1#1	Sense: 5′-GCAGCAUAGAGCUACGCAATT-3′
	Antisense: 5′-UUGCGUAGCUCUAUGCUGCTT-3′
siTIE1#2	Sense: 5′-GGUUACUUGUAUAUCGCUATT-3′
	Antisense: 5′-UAGCGAUAUACAAGUAACCTT -3′
siTIE1#3	Sense: 5′-CGAUGAAGUGUACGAGCUGAU-3′
	Antisense: 5′-AUCAGCUCGUACACUUCAUCG-3′
siBasigin#1	Sense: 5′-GAAGUCGUCAGAACACAUCAA-3′
	Antisense: 5′-UUGAUGUGUUCUGACGACUUC-3′
siBasigin#2	Sense: 5′-GGUCAGAGCUACACAUUGATT-3′
	Antisense: 5′-UCAAUGUGUAGCUCUGACCTT -3′
siBasigin#3	Sense: 5′-CCAGAAUGACAAAGGCAAGAA-3′
	Antisense: 5′-UUCUUGCCUUUGUCAUUCUGG-3′

Supplementary methods

Transwell assays

For the invasion assay, approximately 4×10^4 cells in 200 μL of serum-free medium were seeded into the upper chamber (Corning, USA), which was precoated with Matrigel (BD, USA), while the lower chamber was filled with 600 μL of medium containing 10% FBS for 48 h. After removing the remaining cells in the upper chamber, the cells were fixed with 4% paraformaldehyde and stained with 0.1% crystal violet. The migration assay was performed in the same way as the invasion assay except that no Matrigel was needed. The number of cells was counted in 5 random visual fields under an inverted microscope at 200× magnification. Each assay was repeated three times independently.

Wound healing assay

Cells were seeded in a six-well plate. Then, we used a 200 μL plastic pipette tip to make a scratch on a highly confluent cell monolayer. Scratches were photographed at the indicated times under an inverted microscope (Olympus, Japan) at 40× magnification and analyzed by ImageJ software. All experiments were performed three times independently.

Co-Immunoprecipitation and Mass Spectrometry (Co-IP/MS)

Cells were lysed on ice for 30 min in IP lysis buffer (Beyotime, China) supplemented with protease inhibitor cocktail (Roche, Switzerland) and phosphatase inhibitor (Beyotime, China), followed by 10 cycles of sonication (50% amplitude, 5 s on/off). Then, the cell lysates were centrifuged at 14,000g for 15 min. Cell debris was discarded, and the supernatant was collected. After protein quantification, the cell lysates were precleared by incubating with 50 μL of protein G agarose beads for 1 h. Small samples of precleared lysates were saved as input. Equal amounts of protein were incubated with a specific antibody or a control antibody of the same species overnight at 4°C. The following day, another 50 μL protein A/G agarose beads were added into the cell lysate with gentle rotation at 4°C for 3 h. For anti-FLAG immunoprecipitation, the cell lysates were mixed with anti-FLAG M2 Affinity Gel directly. The agarose beads were washed with TBST more than 5 times. For western blotting, bound proteins were eluted and denatured by boiling beads in loading buffer at 95°C for 10 min. For LC–MS/MS, beads were stored at -20°C until analysis. To screen out candidates for further

study, we set two filters. (1) Number of missed cleavage sites (Count) ≤ 1 for mass spectrometry quality control; (2) iBAQ value of FLAG/IgG ≥ 2 for excluding nonspecific binding proteins.

GEO Dataset Analysis

We downloaded the GSE9750 dataset from the Gene Expression Omnibus (GEO) repository. To ensure data stability and meet the assumptions of parametric analyses, we applied a logarithmic transformation (log2) to the dataset to stabilize variance. Subsequently, correlation analysis was performed to assess the relationship between TIE1 and MMP2, as well as MMP9 in 33 cervical cancer samples. The data analysis was conducted using GraphPad Prism 9.0 software.
